# Good Relationships, Good Performance: The Mediating Role of Psychological Capital – A Three-Wave Study Among Students

**DOI:** 10.3389/fpsyg.2019.00306

**Published:** 2019-02-26

**Authors:** Marcos Carmona-Halty, Wilmar B. Schaufeli, Marisa Salanova

**Affiliations:** ^1^ Escuela de Psicología y Filosofía, Universidad de Tarapacá, Arica, Chile; ^2^ Research Unit of Occupational & Organisational Psychology and Professional Learning, KU Leuven, Leuven, Belgium; ^3^ Department of Clinical Psychology, Utrecht University, Utrecht, Netherlands; ^4^ WANT Research Team, Universitat Jaume I, Castellón de la Plana, Spain

**Keywords:** teacher-student relationships, academic psychological capital, PsyCap, academic performance, high school students

## Abstract

Academic Psychological Capital, or PsyCap, – a set of positive psychological resources encompassing hope, efficacy, resilience, and optimism – has begun to gain attention in academia, showing positive relationships with desirable academic outcomes. However, studies in the field have paid limited attention to the social factors that may increase PsyCap and therefore may lead to positive outcomes. In the present study, we examine whether academic PsyCap mediates between teacher-student relationships and academic performance as assessed by student’s GPA, using a three-wave longitudinal design. Through structural equation modeling, as expected, a statistically significant indirect effect was found between teacher-student relationships and academic performance *via* academic PsyCap. Theoretical and practical implications of the results are discussed; strengths and weaknesses are mentioned; and future research directions are proposed.

## Introduction

Despite previous studies showing positive relationships between academic PsyCap and desirable academic outcomes (e.g., academic adjustment – Liran and Miller, 2017; study engagement – Siu et al., 2014; satisfaction with life – Riolli et al., 2012; and academic performance – Luthans et al., 2012), there are no previous studies of the social factors that might play a role in sparking this positive relationship. This is an important limitation because – based on Goldstein (1999) – academic settings are considered a relational zone in which the quality of the interactions promotes students’ motivation, wellbeing, and performance (Furrer and Skinner, 2003). Thus, providing empirical evidence about the interaction between teacher-student relationships (TSR), academic PsyCap, and academic performance (AP) could be an important step in understanding the role of social factors in building students’ PsyCap and in developing future evidence-based interventions programs to foster students’ PsyCap, wellbeing, and performance.

Self-determination theory (SDT) recognizes the importance of feeling connected to others as a basic psychological need (the so-called need for relatedness) and is considered a fundamental ingredient for functioning at optimal levels (Ryan and Deci, 2000, 2017). Hence, a student who perceives an emotional connection with his/her social environment, believes that s/he is cared for and loved, and feels special to his/her key social partners (e.g., teacher-student relationships) has satisfied his/her need for relatedness. Along this line, previous research has demonstrated the relevant role that interpersonal relationships play in students’ success in terms of engagement, achievement, and wellbeing (Furrer and Skinner, 2003; Cornelius-White, 2007; Roorda et al., 2011; King, 2015; Datu, 2017). The explanation is that high-quality relationships with significant others provide students with the necessary emotional security to actively explore and effectively deal with their (academic) world (Martin and Dowson, 2009).

The conservation of resources (COR) theory recognizes the importance of accumulating resources in the biological, cognitive, and social domains as a strategy to preserve and foster their health and wellbeing, the so-called resource caravan (Hobfoll, 1989, 2002, 2011; Hobfoll et al., 2018). Thus, a student who accumulates personal resources (e.g., hope, efficacy, resilience, and optimism) is more likely to possess the specific skills and attitudes necessary to meet academic requirements and, therefore, achieve academic success. In line with this reasoning, previous research has identified academic PsyCap as a predictor of academic performance (Luthans et al., 2012; Datu et al., 2016; Ortega-Maldonado and Salanova, 2017; Carmona-Halty et al., 2018). The explanation is that academic PsyCap facilitates the processes necessary for students’ attention, interpretation, and retention of positive and constructive memories that are conducive to wellbeing and good performance (Luthans and Youssef-Morgan, 2017).

Overall, the proposed mediational model assumes that students with high-quality TSR will be in a better position to persevere in their objectives (i.e., have hope), rely on their own abilities (i.e., be efficacious), overcome obstacles (i.e., be resilient), and be optimistic about their future (i.e., feel optimism); in turn, these set of four resources would foster AP. In other words, when students satisfy their need for relatedness, they are more likely to accumulate personal resources in the form of PsyCap that can help them to achieve good academic performance. Hence, we tested a structural equation model that assumes that Academic PsyCap mediates between TSR and AP.

## Materials and Methods

### Participants

The sample consisted of 771 high school students attending three Chilean educational institutions. Participants ranged in age from 12 to 18 years (*M* = 14.25, *SD* = 1.60), and 51% of the sample was female. Of the 771 students, 18% were 12 years old, 18% were 13 years old, 19% were 14 years old, 21% were 15 years old, 14% were 16 years old, 7% were 17 years old, and 2% were 18 years old at the time of data collection.

### Procedure

This study was part of a project designed to examine the role of non-intellectual variables in academic performance. The school principals, students, and students’ parents granted a written informed consent. The adolescents voluntarily completed a questionnaire twice: once at the end of the regular academic semester (Time 1: TSR) and once 9 weeks later (Time 2: academic PsyCap). In addition, AP was assessed at the end of the next academic semester, 9 weeks later (Time 3: AP). The online data collection was carried out in a group sessions of about 25 students, and we used the Spanish version of the scales because it is the official language. This study was carried out in accordance with the recommendations of Comité Ético y Científico (CEC-UTA) with written informed consent from all subjects in accordance with the Declaration of Helsinki.

### Instruments

At time 1, TSR was measured using the Teacher-Student Relationships Scale (Martin et al., 2007). This scale has four items (e.g., “My teachers give me the help and support I need”) rated on a scale from 1 (*strongly disagree*) to 7 (*strongly agree*).

At time 2, Academic PsyCap was measured using an adaptation of the Psychological Capital Questionnaire (Avey et al., 2011) to the academic context. This questionnaire has 12 items (e.g., “Right now I see myself as being pretty successful in my studies”) rated on a scale from 1 (*strongly disagree*) to 6 (*strongly agree*).

At time 3, AP was assessed using the grade point average (GPA) provided by the educational institutions. The GPA was based on three mandatory subjects in the Chilean education curriculum: math, language, and history. According to the Chilean grading system, GPAs range from 1 (*poor*) to 7 (*excellent*). The three subjects are offered in both semesters (March-June and July-November), with a total of 6 h per week. For the objective of this study, the GPA was included of the end of the semester before the data collection.

### Data Analysis

All data analyses were conducted using JASP 0.9.01 and SPSS AMOS 23. We used maximum likelihood estimation methods, and goodness-of-fit was evaluated using absolute and relative indexes: *χ* ^2^ and normed *χ* ^2^, incremental fit index (IFI), comparative fit index (CFI), root-mean-squared error of approximation (RMSEA) with a confidence interval (90%), and standardized root mean residual (SRMR). To determine the fit of the model, we followed the European Journal of Psychological Assessment (Schweizer, 2010) and previous recommendations (Schreiber et al., 2006). Finally, we tested the statistical significance of the indirect effects by computing the bias-corrected and accelerated method (BCa) around the indirect effect, as obtained from bootstrapping analysis (Williams and MacKinnon, 2008).

## Results

Table 1 shows means, standard deviations, Cronbach’s α and McDonald’s Ω indexes, and Pearson’s correlations among the variables. The internal consistencies obtained for the scales used were good, and the pattern of correlations revealed significant direct relationships for all the measures in our sample.

**Table 1:** Means (*M*), standard deviation (*SD*), α and Ω indexes, and correlations for the study variables (*n* = 771).

	*M*	*SD*	α	Ω	1	2	3
1. Teacher-student relationship (T1)	5.34	1.39	0.893	0.895	–		
2. Academic PsyCap (T2)	4.06	1.00	0.915	0.916	0.382^**^		
3. Academic performance (T3)	5.31	0.76	0.777	0.780	0.162^**^	0.281^**^	–

** p < 0.001.

**Figure 1:**
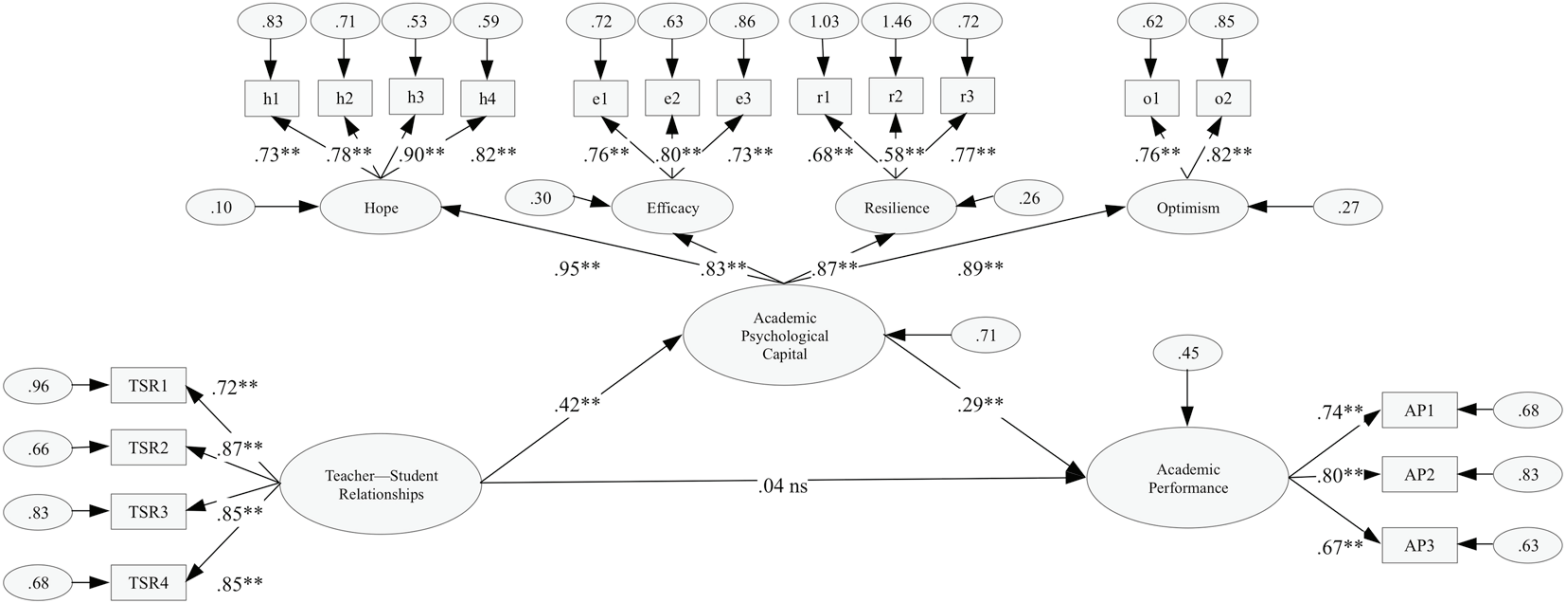
Single mediation model shows the effect of teacher-student relationships on academic performance through academic psychological capital. Standardized coefficients are presented. ** = *p* < 0.001; ns = non-significant effects.

The hypothesized model consisted of 7 latent factors and 19 indicators. That is, the latent TSR one factor reflects TSR with four indicators; academic PsyCap is composed of one-high order factor and four lower-order factors, which, in turn, are formed by 12 indicators, and three indicators make up the latent AP factor. The results showed that this model exceeded the recommended standards and was a good representation of the sample relations, explaining 17.5% of the academic PsyCap variance and 9.8% of the AP variance: *χ^2^* = 694.289; *χ^2^*_/*df (145)*_ = 4.788; *IFI* = 0.929; *CFI* = 0.929; *RMSEA* = 0.070, 90% *CI* (0.065, 0.075); *SRMR* = 0.053. In addition, as Figure 1 shows, the factor loadings were uniformly moderate to high and statistically significant, and exceeded the factor-loading criterion of 0.35 by far (Byrne, 2010).

For the mediation analysis, we implemented a bootstrapping procedure with 5,000 new samples taken from our sample, and indirect effects were calculated (Hayes, 2009). The results led us to conclude that: (1) TSR is significantly related to academic PsyCap [*a* = 0.418, *SE* = 0.039, BCa 95% *CI* (0.339, 0.492), *p* < 0.001]; (2) academic PsyCap is significantly related to AP after controlling for TSR [*b* = 0.295, *SE* = 0.063, BCa 95% *CI* (0.158, 0.403), *p* < .001]; and (3) the indirect effect between TSR and AP – *via* academic PsyCap – is statistically significant [*ab* = 0.123, *SE* = 0.031, BCa 95% *CI* (0.066, 0.186), *p* < 0.001]. In addition, TSR is not significantly related to AP [*c* = 0.039, *SE* = 0.066, BCa 95% *CI* (− 0.082, 0.177), *p* > 0.050]. Hence, we can conclude that academic PsyCap fully mediates the relationship between TSR and AP.

## Discussion

The present study makes an innovative contribution to the scarce research on the antecedents of academic PsyCap (i.e., a set of resources composed of hope, efficacy, resilience, and optimism) and the interactions among TSR, academic PsyCap, and AP.

Consistent with the previous research on SDT – particularly studies on the need for relatedness – we found that students who perceive high-quality relationships with their teachers are more likely to report higher levels of academic PsyCap. In a similar vein, our findings confirmed – in agreement with COR theory – that students who have high levels of academic PsyCap are more likely to achieve better academic performance. Taken together, integrating both SDT and COR theories, we hypothesized and confirmed that academic PsyCap is a mediator between TSR and AP. In other words, we found that the path from good relationships to good performance is fully mediated by academic PsyCap.

The practical implications of the current study are first, rather than focusing exclusively on increasing academic knowledge and skills, teachers should also focus on the affective elements of high-quality relationships with their students; that is, getting along with them, caring about them and showing interest, and providing help and support, among others. According to our results, this focus will help – through increased academic PsyCap – to achieve better academic performance. Second, PsyCap interventions (PCIs) have been carried out by increasing its four components using a within-individual approach (see Luthans et al., 2008; Luthans et al., 2010). However, our results suggest that it might be necessary to include the teachers in an academic PCI as well in order to develop his/her social skills and stimulate developing a high-quality relationship with students. Thus, a between-individual approach could be an important step in future of PCI programs.

The strengths of the current study are first, we used a longitudinal approach, which is scarce in academic PsyCap research; second, we included an objective measure of performance (i.e., GPA); and third, we successfully integrated STD and COR theories in an academic setting. However, there are also some weaknesses that have to be acknowledged. First, to assess TSR and academic PsyCap, we used students’ self-reports. In future research, we could include teachers’ reports about the students’ TSR and their perceptions of their PsyCap. Second, only unidirectional effects were examined (i.e., TSR ➔ academic PsyCap ➔ academic performance). In future research, bidirectional effects using cross-lagged models could be included. Third, only adolescent high school students were recruited. In future research, we could include different academic levels (e.g., undergraduate university students).

Finally, some avenues for future research can be mentioned. First, based on Furrer and Skinner (2003), parent-child and peer-student relationships could be incorporated in our model in order to obtain a better understanding of the role of significant others in academic PsyCap. Second, based on broaden-and-build theory (Fredrikson, 1998), the mediator role of study-related positive emotions between teacher-student relationships and academic PsyCap could be considered. Third, based on Ryan and Deci (2000), basic need satisfaction could be explored as an antecedent of academic PsyCap.

## Data Availability

The datasets generated for this study are available on request to the corresponding author.

## Author Contributions

MCH conceived the idea for the study, conducted the analyses, and wrote the manuscript. MS and WS contributed to the interpretation of results and revised the manuscript.

### Conflict of Interest Statement

The authors declare that the research was conducted in the absence of any commercial or financial relationships that could be construed as a potential conflict of interest.
